# What Are University Professors’ Motivations? A Realistic Approach to Self-Perception of a Group of Spanish University Professors Belonging to the G-9 Group of Universities

**DOI:** 10.3390/ijerph18157976

**Published:** 2021-07-28

**Authors:** Luis Espejo-Antúnez, Mario Corrales-Serrano, Francisco Zamora-Polo, Miguel González-Velasco, María de los Ángeles Cardero-Durán

**Affiliations:** 1 Departamento de Terapeútica Médico Quirúrgica, Facultad de Medicina y Ciencias de la Salud, Universidad de Extremadura, Avda de Elvas s/n, 06006 Badajoz, España; mcarderod@unex.es; 2 Departamento de Didáctica de las Ciencias Sociales, Lengua y Literatura, Facultad de Educación, Universidad de Extremadura, Avda de Elvas s/n, 06006 Badajoz, España; mariocorralesserrano@gmail.com; 3 Departamento de Ingeniería del Diseño, Escuela Politécnica Superior, Universidad de Sevilla, C/Virgen de África, 7, 41011 Sevilla, España; fzpolo@us.es; 4 Departamento de Matemáticas, Universidad de Extremadura, Avda de Elvas s/n, 06006 Badajoz, España; mvelasco@unex.es

**Keywords:** higher education, teachers, motivations

## Abstract

Universities face challenges on a number of levels. In this scenario, university professors play an important role as facilitators of knowledge. The main objective of this study was to analyse the motivations that influence the professional performance in a sample of 102 university professors from nine Spanish public universities (Male: 54 (52.9%); Female: 48 (47.1%)). For this purpose, a questionnaire of 22 closed-ended Likert-type questions was designed, in which scores ranged from 0 to 10 (do not agree at all, strongly agree). Following analysis, the final questionnaire was composed of 17 items, and showed good internal consistency (Cronbach’s alpha = 0.858). The validity analysis showed a value of 0.822 (>0.5) in the sample adequacy measure of Kaiser–Meyer–Olkin and Bartlett’s sphericity test (*p* < 0.0001). The exploratory factor analysis showed a clustering in four factors (two for intrinsic motivations and two for extrinsic motivations), explaining 64.33% of the total variance. Comparisons between each factor score by gender (male and female) showed statistically significant differences for factor F1 (higher for females) and F2 (higher for males). Finally, Q1 and Q13 showed a statistically significant correlation (*p* ≤ 0.05) with years of teaching experience. The motivations of Spanish university professors appear to be associated with the age and gender of the teacher.

## 1. Introduction

University and society are closely related. Although university teaching should be student-centred, university professors play a crucial role in the teaching–learning process [1,2]. In their teaching activity, teachers contribute to the transmission of the specific competences of their disciplines, in addition to a series of transversal competences directly related to the exercise of critical and committed citizenship [1,3,4,5]. In this context, it is necessary to study the attitudes and training required of the teacher for academic performance [6]. Some research in recent decades has focused on changes in teachers’ perceptions [7] and changes in teaching behaviour [8]. However, to the best of our knowledge, teachers’ and students’ motivational variables, as a relevant aspect in academic performance, have not been addressed in depth [9]. Motivation is an essential component in an individual’s formative and professional stages, and determines the effectiveness of the teaching development of university professors [10].

In this sense, the motivational orientations of the teacher in the educational task, in conjunction with the students and in compromise with the educational institution, constitute key factors in the quality of teaching. These factors have been reported as predictors of student learning [9]. However, studies that analyse factors that may be determining motivational orientations in university professors are limited. To the best of our knowledge, this issue has not been addressed in the Spanish university teaching profession [11,12]. According Baumert and Kunter, motivational orientations are related to the “psychological dynamics of behaviour, the maintenance of intentions, and the monitoring and regulation of occupational behaviour” [10]. The complexity of these domains makes it possible to approach teacher motivation as a multidimensional construct from a variety of different approaches. In this sense, previous studies have shown the influence of cognitive (self-efficacy) and affective (enthusiasm) domains of the teacher on the academic performance of students in biology classes [9]. Mahler et al. found a direct relationship between the teacher’s enthusiasm for the subject and for teaching the subject, with the student’s academic performance. By comparison, Kunter et al., 2008 [13], found differences in the quality of teaching behaviour as a function of the interest shown among mathematics teachers (interest in teaching versus interest in the subject). Finally, effort is another factor that plays a role in various motivational theories: for example, it is a relevant factor in both expectancy theory and goal theory [11]. In previous studies, the motivations for using a Learning Management System were studied [14].

As a consequence of the global health crisis caused by the SARS-CoV-2 virus, student and teacher motivation are proving to be key to the development of the teaching and learning process. In recent months, teachers and other professionals in different work environments, in an attempt to give continuity to academic work, have adapted to rapid and unexpected changes. On 14 March 2020, the Government of Spain decreed a state of emergency, thereby beginning a period of confinement to stop the spread of the SARS-CoV-2 virus. This situation forced an immediate adaptation to new educational contexts, in which face-to-face learning was replaced by new methodologies that have been little explored thus far. To cope with this situation in the educational field, the influence of teacher motivation, self-efficacy, and school administrators’ transformational leadership practices on teachers’ innovative behaviour have been highlighted [15,16].

In this sense, this global crisis has underpinned the increasing interest in studying affective (positive and negative) and motivational factors in teachers and students in unpredictable/uncertain environments. However, there are limited studies that analyse the factors that could influence the motivation of university teachers in situations of greater social, economic, and health stability [17,18,19].

Despite the importance of the impact of academics in higher education [2,20,21], studies analysing the interpersonal factors that influence the professional activity of teachers are limited [11,12].

In this paper, the motivation of university teachers is analysed. This is a fundamental result as a proxy indicator of the quality of teaching provided by the educational institution in the field of Higher Education, among many others.

## 2. Theoretical Background

### 2.1. Fundamentals of Motivation

Emotions play a fundamental role in the teaching–learning process. In this sense, the motivation of the professor can contribute to the generation of diverse emotions in the act of teaching [22]. In addition, recent studies state that a teacher’s motivation determines students’ motivation and academic performance [23]. Recently, Skaalvik and Skaalvik found a positive correlation between teachers with low self-efficacy and motivation, and students with stress, dissatisfaction, and misconduct [24]. The effects of university teacher motivation in other competitive spheres have also been observed. Teachers’ motivation has been clearly associated with the degree of interest they show [11]. On the contrary, university professor motivation does not appear to correlate with outcome efficacy and teaching efficacy of the professor [11]. Furthermore, comparisons according to the gender of the task performed have also been made. Thus, women have higher motivations for teaching tasks than men, but no gender differences have been observed for research tasks [25]. Finally, the professional category has also been analysed, in such a way that associate professors and full professors appear to be more motivated in research tasks, and have higher self-efficacy and scientific productivity [25]. There have been limited studies that have delved into the main motivations of university professors. There are two possible causes:

1. Teaching has played a secondary role in the interest of university professors. Traditionally, research activity has been the centre of interest for teachers [23]. Thus, it was assumed that teachers were motivated in their teaching facet.
2. The university professors’ opinion has not been considered a priority in education reforms in recent years.

In Spain, despite the importance of education in the changes of the European Higher Education Area, most of the innovative actions have been linked to the good will of university professors, and there has not been a systematic change in university teaching based on research [26].

### 2.2. Factors Determining the Motivation

From a psychological perspective, motivation has been defined as the process that drives people towards action to achieve a specific goal [27,28,29,30].

From an epistemological perspective, Maslow (1991) explained that motivations are based on human beings’ needs (expressed in pyramidal form) [31]. These needs include biological needs, in addition to anthropological needs such as self-realization. Thus, the motivation comes from [32]:

- The expectations of success in relation to the subjective perception of the probabilities of success in the task (need for power).
- The degree of incentive or challenge involved in a task (need for affiliation).
- Weiner relates motivation to attribution. Attributions influence the expectations of success or failure before a certain task. The attribution of an action can be related to different causes [33].
- Internal or external causes of actions. For example, the teacher’s training preparation may condition his or her attitude or teaching personality, and vice versa.
- Stable or unstable states of the person.
- Controlled or uncontrolled situations.

Recently, from neuropsychology, motivation has been defined as a process in which different mechanisms and neurotransmitters in the brain intervene to activate the human being to achieve an objective, depending on survival instincts or the rational decision to achieve a decided objective [34,35].

Motivation can be considered to be a process. It has an initial phase, in which the person is directed towards the achievement of an action, and a second phase (continuity dimension), which consists of maintaining the effort for the achievement of the task. Authors such as Marina (2013) define these phases as initial motivation and motivation for the task [36].

Ryan and Deci (2017) [37] describe the reasons why a person targets specific objectives using Self-Determination Theory (SDT). These authors describe two “mini-theories” concerning intrinsic motivations (Cognitive Evaluation Theory (CET)) and extrinsic motivations (Organismic Integration Theory (OIT)). These two types of motivations are distinguished by:

- Internal or intrinsic motivation: Intrinsic motivation means that the activity in question is carried out for pleasure or for the satisfaction derived from such activity; for example, when the person expresses his/her interest in the work, thus demonstrating an active role in the achievement of his/her aims, aspirations, and goals.
- External or extrinsic motivation: Extrinsic motivation means that the activity in question is carried out as a means to another outcome or due to a sense of duty; for example, when the advantages offered by the activity in question are taken into account, constituting a means to an end and not an end in itself.

### 2.3. Motivation and the Teaching Profession

The above-mentioned aspects can influence the university professor in a concrete way. University teachers are motivated by various elements, both internally and externally. In the case of the university professor, several types of activities converge teaching, research, and transfer of research results. This work is focused on teaching functions. In this manner, teachers are mobilized towards the exercise of their profession and towards certain teaching objectives [38,39].

As in any other human activity, the decision to engage in the teaching profession, in addition to the performance of that profession, is affected by internal factors, such as vocation or need for personal satisfaction, and external factors, such as family, status, or social recognition. In the case of the teaching profession, motivational factors address the specific features that define the profession [40]. Studies such as that of Burke (1987) categorize the factors that affect the motivation of the teaching profession into two major dimensions (personal and organizational) [41]. Another relevant variable that has been analysed with an impact on teacher motivation is the time spent in the profession [42], or the courses taught [43,44]. However, the number of papers that have studied the teaching motivations of university professors is limited. This paper tries to fill this gap.

### 2.4. Research Objectives

Thus, the aim of the work was to answer the following questions: What are the perceptions of university professors regarding their work, what are their motivations, and are there differences between intrinsic and extrinsic motivations?

Formally, the objectives of this work were: to (1) design a valid and reliable instrument to measure the motivations of Spanish university professors; (2) analyse the relationship between intrinsic or extrinsic motivations with variables such as age, gender, or teaching experience.

## 3. Methodology

### 3.1. Data Collection

This is a descriptive-correlational study based on a cross-sectional study developed through a survey. A questionnaire was designed to determine the teaching motivations of university professors. The questionnaire aimed to categorize the motivations by relating them to intrinsic or extrinsic motivations as proposed by several studies [11,37].

Initially, the questionnaire was provided to a sample of 31 university professors from 9 Spanish universities teaching in various fields of knowledge (scientific, humanistic, biomedical, social, and technical) [39]. Subsequently, the questionnaire was redesigned. For this purpose, improvements were made in the formulation of the questions, and in the inclusion of 1 more item. Then, the questionnaire was analysed by a group of experts. They made a judgement on the comprehensibility of the questions. Finally, the questionnaire was composed of 22 items with Likert answers, ranging from 0 (totally disagree) to 10 (totally agree). Eleven of these were oriented to aspects related to intrinsic motivation as university professors, and 11 were oriented to extrinsic motivation as university professors.

The questionnaire was provided online through the Moodle© virtual platform, This format has advantages as it allows for a quasi-automatic transcription although it may have a lower response rate [45]. Previously, the teachers received information about the nature of the study and its objectives.

### 3.2. Sample Description

A total of 102 university professors voluntarily participated in the study. This study was supervised by the Bioethics Committee of the University of Extremadura, Spain, with ethics approval number 101/2021. Regarding ethical procedures, all participants provided informed consent and gave consent to use their answers for our research with academic purposes. To maintain anonymity, all names were coded. This encouraged professors to freely express their opinion. Inclusion criteria were:

1. Professors (full or part time) from the G9 group of Spanish universities. This group is made up of the following public universities in Spain: Cantabria, Castilla La Mancha, Extremadura, Illes Balears, La Rioja, Navarra, Oviedo, Basque Country, and Zaragoza.
2. Professors with a minimum of two full academic years’ teaching experience at the higher education level.
3. Professors who had access to the Moodle platform of the G9 group of Spanish universities for carrying out at least one activity of the Teaching Training Service during the 2017–18, 2018–19, and 2019–20 academic years.

Professors who did not respond to the questionnaire in the designated period and those who had no employment relationship with the university were excluded.

### 3.3. Data Process

To analyse obtained data, IBM SPSS Statistics software v. 22 for Windows (IBM Corp., Armonk, NY, USA) [46] and the statistical software and programming language R v. 3.6.1 (R Foundation for Statistical Computing, Vienna, Austria) [47] were used.

Firstly, the reliability of the questionnaire was analysed using the Cronbach alpha coefficient [48]. According to studies previously published in the literature, it is considered that a set of items is part of the same construct when an alpha coefficient greater than 0.8 is obtained [49,50]. Successive reliability analyses were carried out to simplify the questionnaire. Previously, the Kaiser–Meyer–Olkin (KMO) sample adequacy measurement [51] and the Bartlett sphericity test [52] were performed to determine whether the study of the dimensional structure of the questionnaire was pertinent.

Secondly, an initial confirmatory factor analysis with the designed questionnaire was analysed to determine if the items could can be grouped into the two dimensions that we initially defined (intrinsic motivations and extrinsic motivations). As this initial analysis was unsuccessful, once the questionnaire was simplified after the successive reliability analyses, an exploratory factor analysis was carried out using principal component analysis as the extraction method, and an oblique rotation method was undertaken (Oblimin with Kaiser normalization) [53,54] to determine the optimal number of dimensions or factors of the new questionnaire.

Subsequently, descriptive analysis of the obtained results was carried out to design a global landscape of the sample. After studying normality [55] and homoscedasticity (Levene’s test) [56], non-parametric inferential analysis was undertaken to identify significant differences between gender (male/female) for the punctuation in the questionnaire using the Mann–Whitney–Wilcoxon test [57], and between ages using the Kruskal–Wallis test [58]. These comparisons were developed at each of three levels: global questionnaire, factors, and each item of the questionnaire.

Finally, the correlation between teaching experience and questionnaire items was analysed using the Spearman correlation coefficient [57] The *p*-values were corrected for multiple tests by the false discovery rate (FDR) method [59].

## 4. Results

The sample presents a homogeneous distribution for the different categories collected (gender, academic position, teaching experience), except age group for the category <30, which represents approximately 4% of the sample (Table 1).

Table 2 shows the statistics for the questions in the questionnaire. The items with the highest scores were Q1, Q4, Q5, Q8, Q10, Q11, and Q21, which all exceeded 8/10 points on the Likert scale (Table 2).

The validity analysis showed a value of 0.786 (>0.5) in the Kaiser–Meyer–Olkin (KMO) sample adequacy measure and Bartlett’s sphericity test (*p* < 0.0001). These results confirm that the analysis is relevant.

The complete questionnaire appears to have good internal consistency, and achieved a large Cronbach’s alpha (0.858). However, the sequential study based on the homogeneity index and the increase in Cronbach’s alpha when each item is eliminated (items whose homogeneity index was less than or equal to 0.2—see Ebel, 1965—and, that when eliminated, Cronbach’s alpha was greater than or equal to that of the total), leads us, by eliminating items Q14, Q18, Q19, Q20, and Q22, to a Cronbach alpha index of 0.872 (Table 3).

Finally, the questionnaire consisted of 17 items (Appendix A). A higher result was obtained in the KMO sample adequacy measure (0.822 (>0.5)) and in Bartlett’s sphericity test, *p* < 0.0001. Thus, the relevance of the analysis was confirmed.

The exploratory factor analysis of the new 17 item questionnaire showed a clustering in four factors—those corresponding with eigenvalues greater than one (two for intrinsic motivation and two for extrinsic motivation)—explaining 64.33% of the total variance (Table 4). The factor analysis according to rotated components showed the following grouping for the four factors **F1:** Q4, Q8, Q9, Q11, and Q21; **F2:** Q13, Q15, Q16, andQ17; **F3:** Q1, Q2, Q3, Q6, Q7, and Q12; **F4:** Q5 and Q10. The items of factors F1 and F4 correspond to intrinsic motivation and the items of factors F2 and F3 correspond to extrinsic motivation. Note that Q1 (Compatible with my values) could be included in both F3 and F4; this is due to the fact that this question shares characteristics of both intrinsic and extrinsic motivation (Table 5).

In general, comparisons made between questionnaire scores by gender (male and female) and by age group (under 30, 30–40, 40–50, and ≥50 years) showed no statistically significant differences (*p* > 0.05) for the total score (i.e., the sum of each item score). However, analysing the total score for each factor (i.e., the sum of the scores of each item of the factor), we found statistically significant differences by gender for factor F1 (higher for females) and F2 (higher for males) (Table 6). No statistically significant differences were found when we compared each factor score by age group (Table 7).

When we analysed each item separately, some of the items showed statistically significant differences (*p* ≤ 0.05); specifically, Q8 and Q13 in the comparison with respect to gender (Table 6) and Q3 and Q7 in relation to age (Table 7).

Finally, regarding the correlation with years of teaching experience, only Q1 and Q13 show a statistically significant correlation (*p* ≤ 0.05), maintaining in both cases a negative correlation (the older the professor, the lower the score on the questionnaire items) (Table 8).

## 5. Discussion

The aim of this study was to design a valid and reliable questionnaire to determine university professors’ motivational orientations. Furthermore, the relationship between motivations and other variables such as age and gender were studied.

The results presented in this work are consistent with those shown by Visser-Wijnveen et al. [11]. Traditionally, the motivational dimension in higher education programs has had little relevance. Therefore, instruments to measure teachers’ motivations are required [11]. A strong correlation between the questionnaire items and the factors distributed in intrinsic or extrinsic motivations was found. Seven items (22 initial items) could be grouped into four factors, explaining 64.33% of the variance. Previously published questionnaires, such as that reported by Visser-Wijnveen et al. [11], started from an original 33-item model, where the confirmatory analysis of the final model showed a distribution of 25 items in five factors (“personal efficacy”, “outcome efficacy”, “teaching efficacy”, “effort”, and “interest”); this grouping explained 52% of the variance. In addition, the internal consistency of the questionnaire designed by these authors for Dutch teachers for the Motivation for teaching scale was lower (0.66) than that shown in the present study for all items.

Statistically significant correlations according to gender and age group were obtained.

Previous studies found that, among the main characteristics of academics, commitment to the promotion of sustainable human development stood out as a motivating factor of university teaching staff internal factors, such as vocation or help to students [39,60,61]. However, to the best of our knowledge, this is the first study that undertook an in-depth exploration of the cognitive domain of motivation (intrinsic and extrinsic) in teachers of the Spanish university system.

The results showed that the aspects grouped in factors 1 and 4, related to the intrinsic motivations of the teacher, scored higher (42.408%). The items grouped in F1 appear to have common characteristics of personal projection towards society (Q4: social utility, Q8: profession allows me to help others, Q9: helps me to be a better person, Q11: allows me to improve society, Q21: teaching allows me to help others). The items with a higher score in Table 2 are grouped in the factors related to internal assets (F1 and F4). Authors such as Pontes Pedrajas et al. [62] analysed the importance of the social utility of university knowledge. Other similar studies present the same idea of the impact of the teaching profession on the improvement of society, in the areas of physical education [63] or health professions [64,65].

Regarding factor 4 (Q5: Appropriate competencies, Q10 Vocation), which contributes 6.071% to the percentage of the accumulated variance, Q1 presented a strong clustering in both F3 and F4, although it was higher in F3. This fact explained why it was associated with F3. Compatibility with personal values has a double meaning, being values related to the internal motivations of the university professor (F4) or values related to external motivations (F3) (Table 5). The main differences that group F1 and F4 into two different factors can be found in the fact that F4 refers to personal characteristics (emanating from the university lecturer’s own being), whereas F1 groups together the possible consequences that such characteristics may have for the university lecturer. Related to the vocation factor, Fernández Guayana affirmed the need for the teaching staff to understand the educational task as a vocation for the other (the student), both from the professional level and the ethical level [66]. Zabalza highlighted the need for teachers to combine their specific training with their vocation to train themselves and train others, which are aspects that directly influence the motivations of professors [67].

Extrinsic motivations were categorized into factors 2 and 3. Factor 2 groups issues of a professor’s interpersonal nature (Q13: no wasted curriculum vitae, Q15: social recognition, Q16: attractive university organization, Q17: teaching work well regarded by society). In relation to F3 (social recognition and the external projection of the teacher), the study of Cuesta-Moreno contributes valuable insights into the teaching experience regarding their social recognition: this study highlights the burden and the concern that generates the need to seek prestige and appreciation at the academic level [68], while also demonstrating the demand for public and social recognition of teachers in society. From this perspective, Malinowska draws attention to the assessment made of the teaching profession in society [69].

In comparisons by gender, our results are consistent with those shown by Bailey [25]. There are statistically significant differences in intrinsic and extrinsic motivations as a function of gender. Our results showed for F1 and F2 that women place more importance on the projection towards the society (for helping others) of their profession than men, and men place greater value on the social recognition of being professors than women. Analysing each item, for item 8, significant differences were found between men and women, with women attaching greater importance to the ability of the profession to help others. In contrast, significant differences were found in item 13. Men, on average, place greater importance on not wasting the curriculum (Table 6). This fact could explain the differences observed with respect to the higher inquiry motivation observed in male teachers in Bailey’s study [25].

Considering that the academic projection of a university professor depends to a great extent on his or her research curriculum [70], this could justify the differences in some questions such as (Q13). Future studies with larger sample sizes and in other educational systems are needed to determine the impact of the professor’s gender on the motivational orientations related to greater social and professional recognition.

Through a survey provided to European academics, Lozano and Barreiro-Gen [71] analysed the integration of sustainable development into the curriculum in higher education institutions. According to the survey results, women tend to integrate sustainable development in a more balanced manner. By comparison, academics from the UK, Sweden, and the Netherlands scored more highly than those from other European countries [71]. The fact that the participants in this study were part of the same university system could be a limitation of the study. Future studies are needed to analyse possible differences in the motivations of university professors from different countries.

Regarding comparisons according to age, certain trends were found. Specifically, significant differences were found in three items (Q3, Q7, and Q17). These items are grouped in F2 (Q17) and in F3 (Q3 and Q7), related to external goods. These results appear to indicate that age conditions the concerns of the university professors. Thus, professors with an age range between 30 and 40 years scored more highly than the remaining age groups regarding the possibility offered by the teaching work to access other professional activities beyond teaching (Q3) and others that allow personal growth (Q7). Statistically significant differences were found between professors under 30 years and the remainder with respect to whether the teaching work is well regarded by society (Q17), showing statistically higher average values than the remaining age groups (Table 7). Professors with higher age groups (40–50 years and >50 years) obtained lower scores in the previous items (Q3, Q7, and Q17) linked to the external goods. These results suggest that teacher training in the Spanish university system should not only focus on the acquisition of knowledge, but also on the management of motivational orientations of both teachers and students [72].

## 6. Conclusions

The designed questionnaire appears to be reliable and valid for detecting motivations in university professor staff. The questions were grouped into four factors (two associated with intrinsic motivations and two with extrinsic motivations). The motivations of Spanish university professors appear to be associated with the age and gender of the teacher.

The results presented here are a starting point for the establishment of policies to promote high motivation of university professors in the field of teaching. These actions could be aligned with those previously exposed by Wilkesmann and Schmid (2013), which are aimed at creating an atmosphere conducive to university teaching and include improving the conditions in which classes are taught, having administrative support for the realization of teaching tasks, and the promotion of teaching innovation actions [70]

One of the limitations of the present study is the small number of responses; in future studies we intend to increase the number of people interviewed, and to use the questionnaire for the evaluation of policies for the promotion of teaching in the university context.

## Figures and Tables

**Table 1:** Participants in the study in frequency percentage.

Title	Category	Frequency (%)
Gender	Male	54 (52.9%)
	Female	48 (47.1)
Age (years)	<30	4 (3.9%)
	30–40	40 (39.2%)
	40–50	38 (37.3%)
	>50	20 (19.6%)
Academic Position	Associate Lecturer	22 (21.78%)
	Lecturer	33 (32.67%)
	Senior Lecturer and Professor	24 (23.76%)
	Others	22 (21.78%)

**Table 2:** Descriptive statistics of the questionnaire items.

Variable	$\bar{x}$ ± SD	Median ± IQR	Min-Max
Teaching Experience (years)	12.96 ± 8.12	11 ± 12.25	2–38
**Q1**	8.76 ± 1.40	9 ± 2	5–10
**Q2**	5.09 ± 2.92	5 ± 4	0–10
**Q3**	6.33 ± 3.13	7 ± 5	0–10
**Q4**	8.65 ± 1.68	9 ± 2	0–10
**Q5**	8.48 ± 1.07	9 ± 1	5–10
**Q6**	6.26 ± 2.58	7 ± 3	0–10
**Q7**	7.77 ± 2.40	8 ± 3	0–10
**Q8**	8.51 ± 1.27	9 ± 1	2–10
**Q9**	7.37 ± 2.19	8 ± 3	0–10
**Q10**	8.73 ± 1.34	9 ± 2	4–10
**Q11**	8.14 ± 1.66	8 ± 1	0–10
**Q12**	6.40 ± 2.50	7 ± 3	0–10
**Q13**	3.98 ± 2.88	4 ± 4	0–10
**Q14**	7.33 ± 2.25	8 ± 2	0–10
**Q15**	2.52 ± 2.47	2 ± 4.25	0–9
**Q16**	5.54 ± 2.78	5 ± 4.25	0–10
**Q17**	6.40 ± 2.49	7 ± 3	0–10
**Q18**	7.93 ± 2.3	8.50 ± 2.25	0–10
**Q19**	1.92 ± 2.80	0 ± 4	0–10
**Q20**	1.47 ± 2.49	0 ± 2	0–10
**Q21**	8.27 ± 1.75	9 ± 2.25	0–10
**Q22**	5 ± 3.23	5 ± 6	0–10

$\bar{x}$: Medium; SD: Standard Deviation; IQR: Interquartile Range; Min: Minimum; Max: Maximum.

**Table 3:** Item–Total score statistics.

Item	Corrected Item–Total Score Correlation (Homogeneity Index)	Cronbach’s Alfa without Element
Q1 Compatible with my values	0.606	0.864
Q2 Adequate economic level	0.433	0.870
Q3 Employability	0.643	0.859
Q4 Social utility	0.508	0.866
Q5 Appropriate competencies	0.389	0.870
Q6 Adequate social level	0.616	0.860
Q7 Access to other studies/personal growth projects	0.550	0.863
Q8 It allows to help other people	0.501	0.867
Q9 Better person	0.599	0.861
Q10 Vocation	0.466	0.868
Q11 It allows to improve the society	0.631	0.862
Q12 Success and recognition	0.573	0.862
Q13 Don’t waste the curriculum vitae working outside the university	0.445	0.869
Q15 University social recognition	0.420	0.869
Q16 I like university	0.413	0.870
Q17 The profession is valued positively by society	0.628	0.859
Q21 Teaching allows me to help others	0.410	0.869

**Table 4:** Grouping of items by factors according to the percentage of variance explained.

Factor	Eigenvalues	% of Variance Explained	% of Cumulative Variance Explained
1	6.177	36.337	36.337
2	2.168	12.753	49.090
3	1.558	9.166	58.256
4	1.032	6.071	64.327
5	0.934	5.493	69.819
6	0.833	4.903	74.722
7	0.744	4.374	79.096
8	0.591	3.479	82.576
9	0.560	3.293	85.869
10	0.412	2.421	88.290
11	0.379	2.228	90.519
12	0.358	2.104	92.622
13	0.341	2.005	94.627
14	0.285	1.677	96.304
15	0.248	1.462	97.766
16	0.211	1.244	99.010
17	0.168	0.990	100.000

**Table 5:** Grouping of items by factors according to rotated component matrix ^1^.

Item	Compound			
	1	2	3	4
Q8 It allows to help other people	0.893	-	-	-
Q21 Teaching allows me to help others	0.873	-	-	-
Q11 It allows to improve the society	0.631	-	-	-
Q4 Social utility	0.539	-	-	-
Q9 Better person	0.496	-	-	-
Q13 Don’t waste the curriculum vitae working outside the university	-	0.795	-	-
Q16 I find the university organisation attractive	-	0.791	-	-
Q15 University social recognition	-	0.718	-	-
Q17 The profession is valued positively by society	-	0.506	-	-
Q2 Adequate economic level	-	-	−0.826	-
Q6 Adequate social level	-	-	−0.765	-
Q3 Employability	-	-	−0.735	-
Q12 Success and recognition	-	-	−0.578	-
Q7 Access to other studies/personal growth projects	-	-	−0.577	-
Q1 Compatible with my values	-	-	−0.455	0.416
Q5 Appropriate competencies	-	-	-	0.885
Q10 Vocation	-	-	-	0.689

^1^ Extraction method: principal component analysis rotation method: Oblimin with Kaiser standardization. Rotation converged into 10 iterations.

**Table 6:** Comparison by gender for the total score, each factor, and each item in the questionnaire.

Item	Gender		*p*-Value
	Male (*N* = 54)	Female (*N* = 48)	
	$\bar{x}$ ± SD Median ± IQR	$\bar{x}$ ± SD Median ± IQR	
Total Score	118.80 ± 19.95 118 43 ± 30.25	115.44 ± 23.83 116.5 ± 31.25	0.573
F1	40.17 ± 5.91 40 ± 5.25	41.81± 7.27 43 ± 6	**0.050**
F2	19.98 ± 6.76 19 ± 6.5	16.71 ± 9.12 15 ± 12	**0.026**
F3	41.63 ± 10.90 42 ± 18.25	39.5 ± 11.24 41.5 ± 17.25	0.334
F4	17.02 ± 2.32 18 ± 3	17.42 ± 1.97 18 ± 10	0.486
Q1	8.63 ± 1.50 9 ± 2	8.92 ± 1.29 9 ± 2	0.377
Q2	5.46 ± 2.96 6 ± 5.25	4.67 ± 2.84 5 ± 4	0.136
Q3	6.5 ± 3.03 7 ± 5	6.15 ± 3.25 7 ± 5.75	0.617
Q4	8.59 ± 1.50 9 ± 2	8.71 ± 1.87 9 ± 2	0.365
Q5	8.43 ± 1.11 8.50 ± 1	8.54 ± 1.03 9 ± 1	0.606
Q6	6.70 ± 2.36 7 ± 4	5.77 ± 2.74 6 ± 3	0.078
Q7	7.85 ± 2.23 8 ± 3	7.69 ± 2.60 8 ± 3	0.942
Q8	8.28 ± 1.32 8 ± 1	8.77 ± 1.17 9 ± 2	**0.021**
Q9	7.15 ± 1.99 7.50 ± 1.75	7.63 ± 2.39 8 ± 2	0.080
Q10	8.59 ± 1.47 9 ±2	8.88 ± 1.18 9 ± 2	0.447
Q11	8.11 ± 1.34 8 ± 1	8.17 ± 1.97 9 ± 1	0.277
Q12	6.48 ± 2.28 7 ± 3	6.31 ± 2.75 7 ± 3	0.991
Q13	4.52 ± 2.55 5 ± 3.25	3.38 ± 3.13 3 ± 5	**0.027**
Q15	2.74 ± 2.40 3 ± 4	2.27 ± 2.56 1 ± 5	0.237
Q16	6.09 ± 2.33 6 ± 3	4.92 ± 3.13 5 ± 5	0.059
Q17	6.63 ± 2.02 7 ± 2	6.15 ± 2.93 7 ± 4	0.627
**Q21**	8.04 ± 1.73 8 ± 2	8.54 ± 1.76 9 ± 2	0.062

$\bar{x}$: Medium; SD: Standard Deviation; IQR: Interquartile Range; *p*-value: significance level. Results with *p*-values ≤ 0.05 are shown in bold type.

**Table 7:** Comparison by age for each item in the questionnaire.

Item	Age				*p*-Value
	<30 (*N* = 4)	30–40 (*N* = 40)	40–50 (*N* = 38)	>50 (*N* = 20)	
	$\bar{x}$ ± SD Median ± IQR	$\bar{x}$ ± SD Median ± IQR	$\bar{x}$ ± SD Median ± IQR	$\bar{x}$ ± SD Median ± IQR	
Total Score	123.8 ± 18.46 117 ± 30.75	122.4 ± 20.1 127 ± 31.25	111.8 ± 24.1 111 ± 32.25	115.9 ± 19.8 115 ± 20	0.197
F1	44.5 ± 6.4 45 ± 11.5	40.9 ± 6.48 41.5 ± 7.75	40.03 ± 7.45 40.5 ± 7	42.05 ± 5.04 41 ± 5.5	0.691
F2	23.75 ± 6.18 23 ± 11.25	19.83 ± 7.72 19.5 ± 9.75	17.18 ± 8.40 18 ± 7	17 ± 8.12 17 ± 11.75	0.120
F3	38.25 ± 10.84 37.5 ± 20.75	44.08 ± 9.88 47 ± 17.25	37.84 ± 11.7 37 ± 20.25	39.5 ± 11.13 41 ± 15.5	0.107
F4	17.25 ± 0.96 17.5 ± 1.75	17.58 ± 2.02 18 ± 3	16.73 ± 2.32 17 ± 2.5	17.35 ± 2.23 18 ± 2.75	0.369
Q1	9.25± 0.96 9.50± 1.75	9.15 ± 1.25 10 ± 1	8.39 ± 1.55 9 ± 2	8.60 ± 1.31 8.5 ± 2	0.057
Q2	3.50± 2.89 3.50 ± 5.50	5.05 ± 3.08 5 ± 4.75	5.24 ± 2.76 5.50 ± 4.25	5.20 ± 3 6 ± 5.75	0.698
Q3	6± 1.16 6 ± 2	7.43 ± 2.81 8 ± 3.50	5.47 ± 3.21 6 ± 5.25	5.85 ± 3.35 6 ± 5.75	**0.026**
Q4	9.25± 1.50 10 ± 2.25	8.73 ± 1.62 9 ± 2	8.37 ± 2.02 9 ± 2	8.90 ± 0.97 9 ±2	0.611
Q5	8.25± 0.50 8 ± 0.75	8.75 ± 0.98 9 ± 2	8.26 ± 1.16 8.50 ± 1	8.40 ± 1.10 8.50 ±1	0.315
Q6	5.50± 3 5 ± 5.50	7.03 ± 2.19 7.50 ± 2.75	5.68 ± 2.88 6 ± 3.25	6 ± 2.41 6 ± 3	0.137
Q7	7.25 ± 2.06 7 ± 3.75	8.65 ± 1.70 9 ± 2	7.37 ± 2.59 8 ± 2	6.90 ± 2.85 7.50 ± 4.75	**0.018**
Q8	9 ± 1.41 9.50 ± 2.50	8.43 ± 1.58 9 ± 1.75	8.34 ± 1.10 8 ± 1	8.90 ± 0.72 9 ± 1	0.246
Q9	8.25 ± 2.06 8.50 ± 3.75	7.40 ± 2.36 8 ± 3	7.37 ± 2.09 8 ± 2.25	7.15± 2.16 8 ± 3.75	0.809
Q10	9 ± 1.16 9 ± 2	8.83 ± 1.34 9 ± 2	8.47 ± 1.43 9 ± 1.25	8.95± 1.23 9 ± 1.75	0.473
Q11	9 ± 1.16 9 ± 2	8.15 ± 1.55 8 ± 1	7.79 ± 1.96 8 ± 2	8.60 ± 1.19 9 ± 1.75	0.250
Q12	6.75 ± 2.63 7.50 ± 4.75	6.78 ± 2.53 7.50 ± 3.50	5.68 ± 2.61 6 ± 4.25	6.95± 2.04 7 ± 3	0.176
Q13	5.75 ± 2.50 5.50 ± 4.75	4.53 ± 2.92 4.50 ± 4.75	3.47 ± 2.94 4 ± 5.25	3.50 ± 2.61 3± 3.75	0.210
Q15	2.25 ± 1.50 3 ± 2.25	2.60 ± 2.32 2 ± 5	2.58 ± 2.83 2 ± 5	2.30 ± 2.34 2.50± 3	0.957
Q16	7.25 ± 3.40 8 ± 6.25	5.78 ± 2.79 5.50 ± 2.75	5.24 ± 2.74 5 ± 5	5.30 ± 2.81 5 ± 5	0.528
Q17	8.50 ± 1.29 8.50 ± 2.50	6.93 ± 2.42 7.50 ± 2.75	5.89 ± 2.61 6.5 ±4	5.90 ± 2.25 6.50 ± 3	**0.044**
Q21	9 ± 1.41 9.50 ± 2.50	8.20 ± 1.95 9 ± 2.75	8.16 ± 1.81 8 ± 2.25	8.50 ± 1.28 9 ± 1	0.707

$\bar{x}$: Medium; SD: Standard Deviation; IQR: Interquartile Range; *p*-value: significance level. Results with *p*-values ≤ 0.05 are shown in bold type.

**Table 8:** Correlation between professors’ years of experience and the questionnaire items.

Item	Spearman Rho Correlation Coefficient	Rho *p*-Value	Corrected *p*-Value
Q1	−0.293	0.003	0.024
Q2	0.102	0.307	0.435
Q3	−0.174	0.080	0.271
Q4	−0.116	0.288	0.435
Q5	−0.111	0.268	0.435
Q6	−0.141	0.156	0.380
Q7	−0.145	0.146	0.380
Q8	−0.027	0.785	0.953
Q9	−0.125	0.210	0.397
Q10	0.005	0.962	0.975
Q11	−0.090	0.370	0.484
Q12	−0.134	0.181	0.384
Q13	−0.302	**0.002**	**0.024**
Q15	−0.003	0.975	0.975
Q16	−0.191	0.055	0.232
Q17	−0.201	0.042	0.232
Q21	−0.010	0.924	0.975

Results with *p*-values ≤ 0.05 are shown in bold type.

## Data Availability

The data presented in this study are available on request from the corresponding author (Luis Espejo-Antúnez: luisea@unex.es).

## Appendix A

**Table A1:** Cuestionario Sobre Motivaciones del Profesorado Universitario.

**Género:** ☐ Mujer ☐ Hombre
**Age:**
** ☐ Menor 30 años ☐ De 30 y 40 años ☐ De 40 y 50 años ☐ Más de 50 años**
**Centro donde impartes docencia:…………………………………………………**
** ☐** Profesor Asociado
☐ Profesor Ayudante
Profesor Contratado Doctor
☐ Profesor Titular o Catedrático
☐ Otros
**Experiencia y carga docente actual (Nºaños/créditos):…………………………………………………………………………**
**Valora la influencia que tienen cada uno de los siguientes ítems en tu ejercicio como profesor universitario. Indica el grado de acuerdo en cada ítem, siendo “0” nada de acuerdo y “10” totalmente de acuerdo**
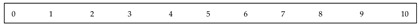
**Es una profesión compatible con mis valores** **La profesión docente para la que me preparé está bien pagada** **El ser profesor universitario me puede permitir acceder a otras actividades profesionales más allá de la docencia.** **Es una profesión con utilidad social** **Se trata de una profesión para la que tengo capacidades y aptitudes** **La profesión puede aportarme un buen estatus social** **El ser docente universitario me permite acceder a otros estudios/proyectos de crecimiento personal** **La profesión me permite ayudar a los demás** **El ser docente universitario me ayuda a ser mejor persona** **Es una profesión por la que siento vocación** **La profesión de docente universitario me da la posibilidad de trabajar con otros para mejorar la sociedad** **Puedo tener éxito y reconocimiento impartiendo docencia en la titulación para la que me preparé en un inicio** **No quise desperdiciar mi curriculum para optar a un puesto fuera de la universidad** **Consideré que mi trabajo docente iba a estar relacionado con la titulación para la que me formé y especialicé** **Soy docente para obtener un reconocimiento social universitario, aunque no esté relacionado con la profesión a la que deseo acceder o ejercer** **Me pareció atractivo el tipo de organización universitaria, en la que siempre quise trabajar** **La labor docente para la que me he preparado y sigo preparando está bien vista por la sociedad** **La profesión del docente universitario es creativa** **La tradición familiar ha motivado mi decisión de ser docente universitario** **Mi realidad como docente universitario existe porque no pude acceder a los estudios o salidas profesionales que verdaderamente deseaba** **La enseñanza me permite ayudar a otros** **Doy clases de las asignaturas impartidas porque fueron las que más me gustaban cuando cursaba estos contenidos en la titulación universitaria**
